# In situ Thermoreflectance Characterization of Thermal Resistance in Multilayer Electronics Packaging

**DOI:** 10.1021/acsaelm.1c01239

**Published:** 2022-03-24

**Authors:** Nathawat Poopakdee, Zeina Abdallah, James W. Pomeroy, Martin Kuball

**Affiliations:** Center for Device Thermography and Reliability (CDTR), University of Bristol, Bristol BS8 1TL, United Kingdom

**Keywords:** frequency-domain thermoreflectance, thermal resistance, thermal conductivity, die attach, packaged device, thermal management, reliability

## Abstract

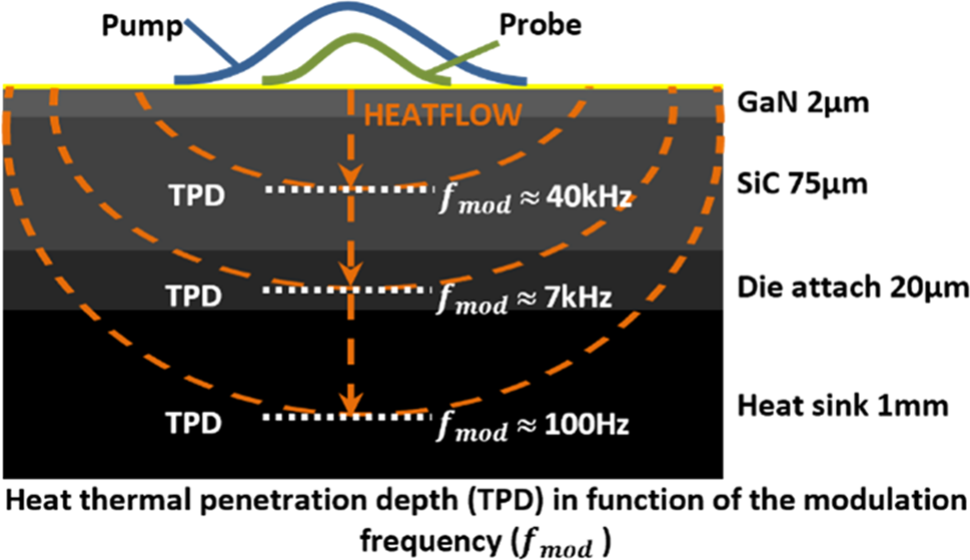

High-performance, high-reliability microelectronic devices are essential for many applications. Thermal management is required to ensure that the temperature of semiconductor devices remains in a safe operating range. Advanced materials, such as silver-sintered die attach (the bond layer between the semiconductor die and the heat sink) and metal-diamond composite heat sinks, are being developed for this purpose. These are typically multilayered structures, with individual layer thicknesses ranging from tens of micrometers to millimeters. The effective thermal conductivity of individual layers likely differs from their bulk values due to interface effects and potential material imperfections. A method is needed to characterize the thermal resistance of these structures at the design optimization stage to understand what effect non-idealities may have on the final packaged device temperature. We have adapted the frequency-domain thermoreflectance technique to measure at low frequencies, from 10 Hz to 10 kHz, enabling multiple layers to be probed at depths from tens of micrometers to millimeters, which is tailored to assess novel device packaging and heat sinks. This is demonstrated by measuring the thermal resistance of a sintered silver die attach.

## Introduction

The demand for increased performance and increased efficiency has led to more compact, higher power density electronic and optoelectronic semiconductor devices. Dissipating waste heat efficiently is increasingly challenging in devices such as laser diodes^1^ and GaN RF HEMTs,^2^ a trend that will continue. For example, GaN-on-diamond transistors will be operated at three times the areal power density of the current generation GaN-on-SiC transistors in RF applications;^3^ GaN-on-SiC operating power is already known to be limited by the thermal resistance of conventional packaging (die attach and flange material thermal conductivities). Excessively high operating temperatures degrade the performance and reliability of electronic devices. This has motivated the development of advanced low thermal resistance electronic packaging materials, such as thermal interface materials (TIM) and heat sinks, which are needed to reach the full potential of high-performance electronic devices. Examples of advanced device packaging materials include sintered silver die attach,^4^ copper-molybdenum copper (CMC),^5^ diamond,^6^ and metal-diamond composites.^7^ Measuring the thermal properties of individual material layers and their interfacial thermal resistances, e.g., the die attach versus the flange, is crucial both for material development and to accurately predict device operating temperatures. The measurement of thin, high thermal conductivity diamond heat spreaders is equally important but technically challenging for laser flash analysis (LFA).

Various existing techniques are used to measure the thermal properties of materials.^8^ Macroscopic, uniform samples can be measured using the hot bar or disc method.^9^ LFA measures depth-averaged thermal diffusivity, with limited sensitivity to the thermal properties of individual layers.^10^ Conventional laser flash cannot easily measure thin high thermal conductivity materials with high enough accuracy, such as a ∼100 μm-thick diamond heat spreader with a thermal conductivity ≥1000 W/m·K. Therefore, there is an increasing need for a technique capable of measuring high thermal conductivity materials as well as multilayer structures. Conventional time-domain thermoreflectance (TDTR), and frequency domain thermoreflectance (FDTR) are well-established techniques for measuring thermal properties of a wide range of materials, including “bulk” materials, thin films,^11^ superlattices, and nanolaminates.^12^ Conventional TDTR and FDTR provide high lateral spatial resolution but cannot probe depths greater than tens of micrometers^13−16^ because of small pump sizes. Therefore, the properties of thicker multilayer electronics materials, including those buried under other layers, cannot be measured, for example, die attach and packaging materials beneath a microchip. A novel steady-state thermoreflectance (SSTR) technique has been demonstrated to measure a wide range of thermal conductivities (1–2000 W/m·K).^17,18^ The SSTR technique is based on a single low modulation frequency (<1 kHz), i.e., quasi-steady-state heating. The advantage of this technique is that the depth probed is similar to the pump laser spot size, and so a depth of several micrometers to hundreds of micrometers can be achieved by tuning the spot size. However, precise adjustment of the pump spot size is practically more difficult to achieve than the modulation frequency sweep used for low-frequency FDTR presented here, which can probe continuously variable depths through a multilayer structure.

We have adapted the FDTR method to lower modulation frequencies (less than 10 kHz) tailored to measure multiple layers of thicknesses ranging from tens of micrometers to several millimeters. We demonstrate the effectiveness of this technique by measuring individual layers of a packaged semiconductor device in situ, which could previously not be measured with high accuracy before device fabrication. Packaged devices have been measured using frequency domain electrical heating/thermometry,^19^ which requires extensive fabrication of electrical heaters and thermal sensors. In contrast, the low-frequency FDTR method presented here requires only minimal sample preparation, i.e., transducer layer deposition, and can be done quickly during development or for process control. Thermal conductivity measurement accuracy is assessed by measuring a range of materials with well-known thermal properties, including through a 0.25 mm-thick, 1000 W/m·K diamond heat spreader, which is challenging to measure using LFA. Finite element simulations are used to show how improving the thermal resistance characterization accuracy of multilayer structures impacts device channel temperature predictions.

The FDTR method is based on an optical pump–probe configuration, which uses a frequency-modulated pump laser to periodically heat the sample’s surface, typically coated with a metal transducer. A probe laser, usually with a different wavelength to the pump, is used to monitor the surface temperature change Δ*T* of the transducer as it is proportional to the relative change in reflectivity Δ*R* of the transducer, Δ*R*/*R* ∝ Δ*T.*^20^ The reflected probe beam is detected by a photodetector, and the signal is measured using a lock-in amplifier. The phase lag of the probe signal with respect to the reference signal varies with modulation frequency, which is analyzed using a heat diffusion model to determine the thermal properties of the sample.

**Figure 1:**
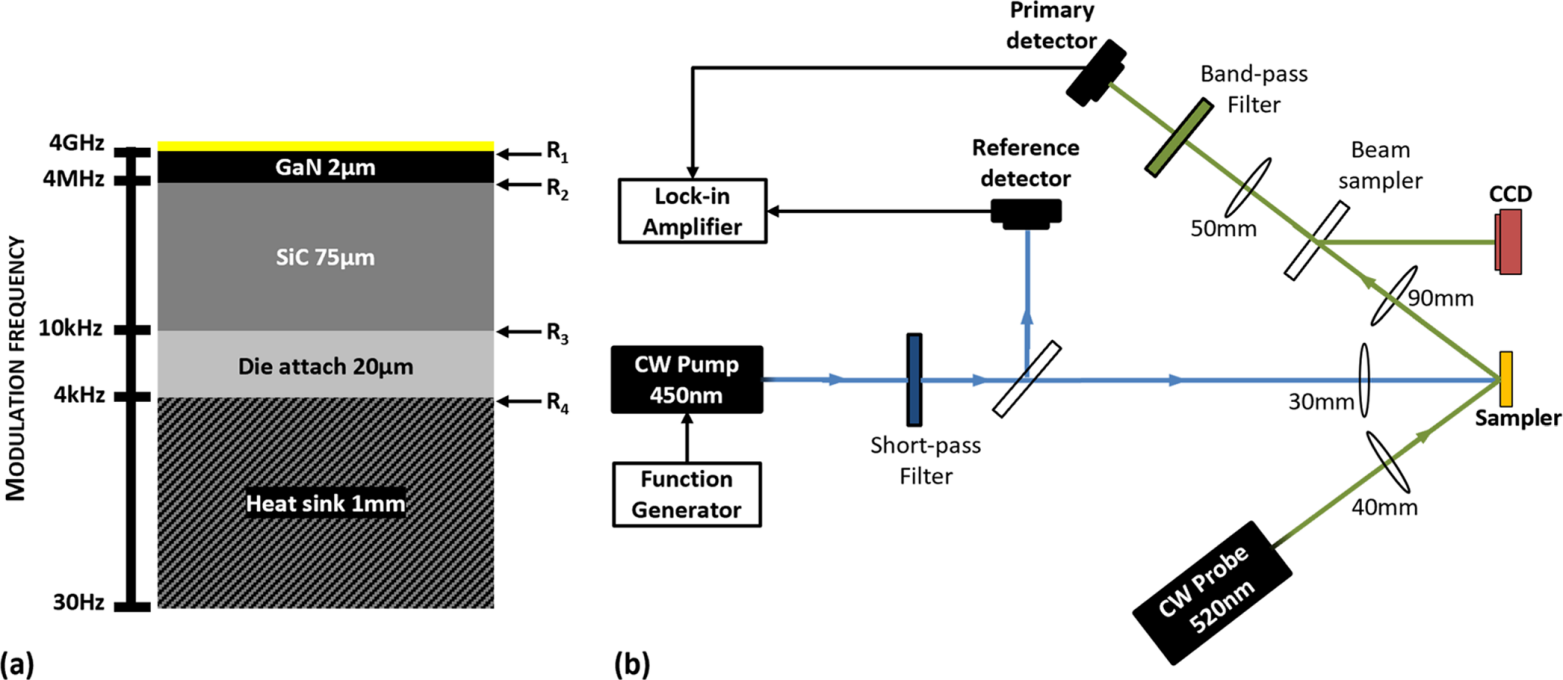
(a) Schematic of packaged GaN-on-SiC transistor illustrating the depth of the heat at different modulation frequencies. *R*_1_, *R*_2_, *R*_3_, and *R*_4_ represent the thermal boundary resistances at each interface. (b) Schematic showing the main components of the low-frequency FDTR system.

As mentioned previously, one of the major advantages of FDTR is the ability to adjust the depth probed. The thermal penetration depth (TPD) of FDTR measurements can be adjusted by changing the modulation frequency *f* of the pump beam. For one-dimensional (1D) heat diffusion, , where κ_*z*_, *C* are the cross-plane thermal conductivity and the volumetric heat capacity of the sample,^21^ respectively; this is only valid when TPD ≪ the pump spot radius, i.e., in the high-frequency limit. This feature makes the FDTR method suitable for the measurement of multilayer samples such as packaged electronic devices. Figure 1a shows the structure measured in this work, where the probing depths corresponding to different modulation frequencies are indicated. However, this type of structure cannot be measured using conventional FDTR^13,14,22^ because the small pump beam diameter used in these setups limits the thermal penetration depth. Therefore, a larger pump spot size is essential to enable greater depths to be probed at lower modulation frequencies.^21^ Increasing the pump spot size, however, will reduce the incident pump laser power density, reducing the Δ*T* and the signal generated. In the low-frequency regime, the measured signal is therefore easily overwhelmed by 1/*f* noise;^23^ other sources of environmental noise also increase at low frequency. These effects may be compounded by a high thermal conductivity of the materials, which are of interest for packaging thermal management applications, which further reduces the Δ*T* induced for given laser power. Hence, a larger pump beam radius and higher laser power, in combination with minimizing parasitic sources of noise, are required to extend the depth probed by FDTR from tens of micrometers to millimeters.

## Experimental Setup

Figure 1b shows a schematic of the developed FDTR system, similar to the pump–probe configuration of Schmidt et al.^13^ A ∼7 W maximum output power 450 nm laser diode (pump) is modulated by a function generator via a current driver to provide periodic heating at a frequency between 1 Hz and 10 kHz. A 520 nm green laser of ∼110 mW maximum output power is used as a probe in our setup. A 500 nm cut-off frequency short-pass filter is placed after the pump laser, blocking any emission at the probe laser wavelength. The pump beam is focused on the sample through a 30mm focal length lens (NA ≈ 0.3). The maximum achievable continuous-wave (CW) laser power impinging on the sample is over 1.4 W, with a close to Gaussian beam profile, which was checked using a beam profiler.

To accurately measure the reflected phase of the probe beam, the frequency-dependent phase shift introduced by various instrumentation components, ϕ_instr_, must be eliminated.^24^ To achieve this in our low-frequency FDTR setup, a glass slide is used to reflect a portion of the pump beam into a reference photodiode detector, which is used as the lock-in amplifier reference signal, canceling ϕ_instr_. It should be noted that this scheme is suitable for low-frequency FDTR, but other contributions to ϕ_instr_ need to be considered for high-frequency measurements, i.e., at > MHz frequencies, the instrument phase shift caused by the probe and reference optical path length difference must be accounted for; this is negligible for the low modulation frequencies used here, e.g., our highest modulation frequency, 10 kHz, only produces a phase shift of about 0.0001° per cm. This low-frequency FDTR system is simpler and lower cost than typical high-frequency FDTR instruments. For example, a simple optical arrangement is used, where the probe beam was focused on the sample at ∼10° angle of incidence, whereas the pump beam is at normal incidence, avoiding the need for combining the beams. Keeping the pump and probe beam paths separate reduces complexity, but it should be noted that this approach would be unsuitable for high-frequency measurements for the optical delay reason already discussed.

The pump spot radius is an important parameter because it strongly affects the measured phase. To accurately calibrate the pump spot size and to consider that the beam profile is not perfectly Gaussian, a high purity silicon sample with precisely known thermal properties has been used as a reference sample; the effective pump spot radius is determined as a fitting parameter. The typical probe spot radius is ∼20 μm, which is 30× smaller than the pump spot radius (compared and measured by a CCD camera), reducing the effect of probe beam walk-off resulting from beam misalignment.^25^

The samples were coated with a 10 nm chromium (Cr) adhesion layer and a 150 nm gold (Au) transducer layer,^22,26,27^ using thermal evaporation prior to the FDTR measurements; note that low-frequency measurements are less sensitive to the thermal properties of the transducer than conventional high-frequency FDTR. Approximately 60% of the 450 nm pump beam power is absorbed, heating the transducer.^22^ The reflectivity change of the transducer, which is proportional to the change in the surface temperature, is monitored by the reflected probe beam intensity. The high thermoreflectance coefficient of gold (*C*_TR_ = 2.3 × 10^–4^ K^–1^) at the chosen 520 nm probe wavelength ensures a high measurement sensitivity.^28^

The reflected probe beam is collimated by a 90 mm focal length lens and focused onto the primary detector by a 50 mm focal length lens. Note that the primary and reference photodetectors are identical. A bandpass filter is used to prevent scattered pump light from reaching the primary detector. A beam sampler is used to direct a portion of the probe beam onto a CCD camera, which is used for viewing the probe beam on the sample, aiding focusing.

The primary and reference detector signals are inputted into the lock-in amplifier, which measures the phase shift of the probe signal with respect to the reference signal as a function of modulation frequency. Phase noise and the uncertainty in controlled parameters, e.g., spot size, thickness, and specific heat capacity, are the main sources of uncertainty in the thermal model. The phase noise was obtained from the standard deviation of 10 repeated phase measurements, with a duration of ∼1 min per measurement.

## Heat Diffusion Model

The solution of heat conduction in the frequency domain for a multilayer system has been well established and explained in several works.^11,13,22,29,30^ Here, we summarize the key features crucial to understanding our analysis. In FDTR, the surface is heated periodically by a Gaussian pump beam with a 1/e^2^ spot radius of *w*_0_. The thermal response is monitored by a Gaussian probe beam with a 1/e^2^ spot radius of *w*_1_. Considering the heat conduction in a uniform slab of material, the temperature *T*_t_ and heat flux *F*_t_ on the top surface are related to temperature *T*_b_ and the heat flux *f*_b_ on the bottom surface by
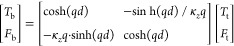
1with
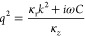
2where *d* is the thickness, κ_*z*_ and *κ*_r_ are the cross-plane and in-plane thermal conductivities, respectively. *C* is the volumetric heat capacity, ω is the modulation frequency, and *k* is the Hankel transform variable.^31^

For a multilayer structure of *n* layers with the thermal boundary resistance *R* between individual layers, the matrices *M* for individual layers are multiplied in sequence. The temperature *T*_b_*n*__ and heat flux *F*_b_*n*__ on the bottom surface of the bottom layer are given by
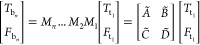
3where *T*_t_1__ and *F*_t_1__ are the temperature and heat flux on the top surface of the top layer, respectively. The thermal boundary resistance *R* is treated by taking the limit as the volumetric heat capacity *C* → 0 and choosing κ_*z*_ and *d* such that *R* = d/κ_*z*_.^13^ If the final layer is considered as semi-infinite or an adiabatic boundary condition is assumed for the bottom surface, then *F*_b_*n*__ = 0 and T_(t_1_)_ = −D̃/C̃*F*_(t_1_)_^13^ For a Gaussian pump beam, the heat flux *F*_*t*_ on the top surface of the sample in the Hankel transform space is given by
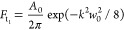
4where *A*_0_ is the absorbed pump laser power.

Finally, the thermal response in the frequency domain weighted by the Gaussian probe beam^11^ is given by
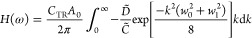
5where *C*_TR_ is the thermoreflectance coefficient. The phase lag of the probe signal measured by the lock-in amplifier Δϕ is given by^32^
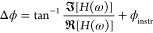
6where ϕ_instr_ is the total frequency-dependent instrument phase shift of all components, which is canceled using a reference detector, as previously mentioned.

Unknown thermal properties, including thermal conductivities and thermal boundary resistances, are determined by nonlinear least-squares fitting, which minimizes the error between the measured phase data from the lock-in amplifier and the thermal model.

The analytical variance–covariance matrix method from Yang et al. was used to estimate the uncertainty in the fitted parameters (± standard deviation)^33^
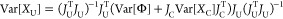
7where Var[*X*_U_], Var[*X*_C_], and Var[ϕ] are the covariance matrices of the unknown parameter vector *X*_U_, the controlled parameter vector *X*_C_, and the phase noise ϕ, respectively. *J*_U_ and *J*_C_ are the Jacobian matrices of the unknown parameter and controlled parameter, respectively. The standard deviations of the fitted parameters are obtained by taking the square root of the diagonal elements of Var[*X*_U_].

## Results and Discussion

To ensure the thermal conductivity measurement accuracy of the new instrument, we initially performed benchmark measurements of standard materials with known thermal conductivities spanning an order of magnitude: sapphire, silicon, copper, and polycrystalline diamond (Element Six TM100).

### Sensitivity Analysis

A sensitivity analysis was performed using the material properties given in Table 1; this is a key step to determining which thermal properties we can measure with high confidence and at what frequency range. We use the same approach as that of Schmidt et al.^13^ to calculate the phase sensitivity *S*_*x*_ to a measurement parameter *x*.
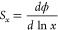
8where ϕ is the phase response of the thermal model in radians. Typical parameters of interest are thermal conductivity κ and thermal boundary resistance *R* between the transducer and the sample. A sensitivity plot indicates the dependency of the phase signal on the change in that specific thermal property and the accuracy with which this property can be measured.

**Table 1:** Properties of Test Samples Used for System Validation, Including Thickness (d), Thermal Conductivity (κ), Density (ρ), and Specific Heat Capacity (c_p_)

material	*d* (mm)	κ (W/m·K)	ρ (g/cm^3^)	*c*_p_ (J/g·K)
sapphire	5	40^a^	3.980	0.773 ± 0.003^34^
silicon	5	149^35^	2.329^35^	0.713 ± 0.004^36^
copper	2.89	390^35^	8.940^35^	0.386 ± 0.008^36^
TM100^b^	0.25	1000	3.520	0.520

a www.azom.com: Sapphire single crystal.

b The properties of TM100 were obtained from the manufacturer.

**Figure 2:**
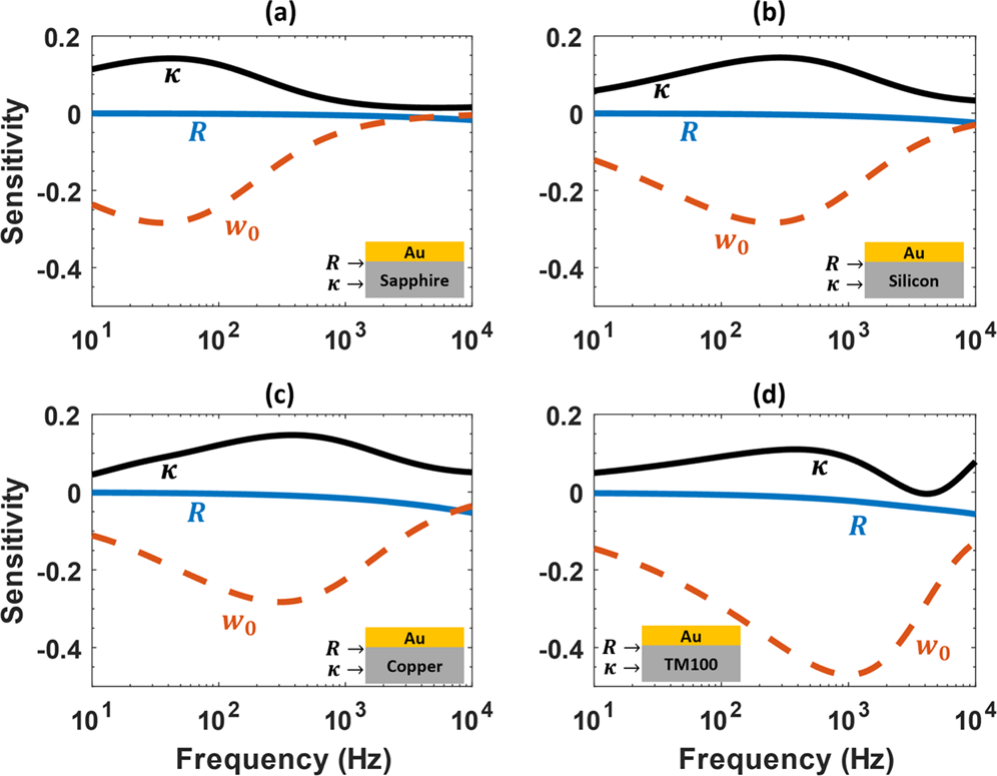
Phase sensitivity to the pump spot radius *w*_0_, the thermal conductivity κ, and thermal boundary resistance *R* between the 150 nm gold film and substrate for (a) 5 mm-thick sapphire, (b) 5 mm-thick silicon, (c) 3 mm-thick copper, and (d) 0.25 mm-thick TM100 samples.

Figure 2 shows the phase sensitivity for the samples from Table 1. A pump spot radius of 580 μm measured by CCD camera is considered in the sensitivity study and a thermal boundary resistance *R* = 1 × 10^–8^ m^2^·K/W is assumed between the 150 nm Au transducer and the substrate for all samples.^37^ For the sapphire sample, the thermal conductivity κ dominates the measurement at frequencies lower than 1 kHz, and the sensitivity to *R* increases only slightly at higher frequencies. The thermal conductivity of the silicon and copper samples has a similar frequency response. In contrast, for the higher thermal conductivity TM100 sample, the measurement is most sensitive to κ around 1 kHz. The sensitivity to *R* shifts to lower frequencies for higher thermal conductivity materials.

### Pump Spot Size Calibration

Pump spot size is an important factor in the FDTR measurements. The phase sensitivity to the pump spot radius *w*_0_ is shown in Figure 2. For sapphire, silicon, and copper, the peak sensitivity to the spot radius is ∼2× higher than the peak sensitivity to the thermal conductivity. However, for the TM100 sample, the peak sensitivity to the spot radius is ∼4× higher than the peak sensitivity to the thermal conductivity. This implies, on the one hand, that for a thick sample, the spot radius sensitivity peaks at a modulation frequency where *w*_0_ ≈ 2 × TPD, and on the other hand that the sensitivity increases when the thermal penetration depth exceeds the sample thickness, e.g., the case of thin TM100 sample. It is noteworthy at this stage that since the probe spot radius is 30× smaller than the pump spot radius, the effect of probe radius uncertainty on the thermal response is negligible.

**Figure 3:**
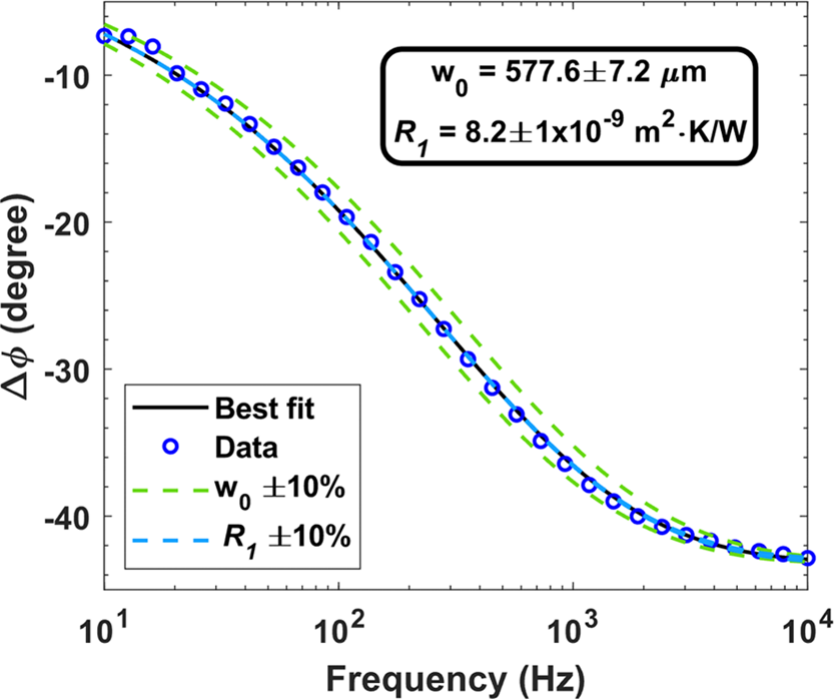
Measured FDTR phase data of a 5mm silicon with the best-fit curve. Dashed curves are obtained by varying the best-fit values ±10%. Inset: fitted spot radius *w*_0_ and thermal boundary resistance *R*_1_ at the transducer/silicon interface parameters.

A reference silicon sample has been used to fit the effective pump spot radius; this was found to be more accurate than a direct beam profiler measurement due to the slight non-ideality of the Gaussian beam profile. Figure 3 shows the silicon measurement results and fit, where the known thermal properties (Table 1) are fixed, but *w*_0_ and *R*_1_ have been adjusted to best fit the measured phase curve; as previously discussed, the contribution from *R*_1_ is insignificant for silicon. Using the silicon properties in Table 1, the pump spot radius and the thermal boundary resistance at the transducer and silicon interface have been determined. The extracted pump spot radius is 577.6 ± 7 μm, and the thermal boundary resistance is 8.2 ± 1 × 10^–9^ m^2^·K/W. The error bars for both extracted parameters are related to the measurement’s phase noise. The fitted spot radius is subsequently used to determine the thermal conductivity and the thermal boundary resistance of the other samples investigated here.

### Verification Measurements

**Figure 4:**
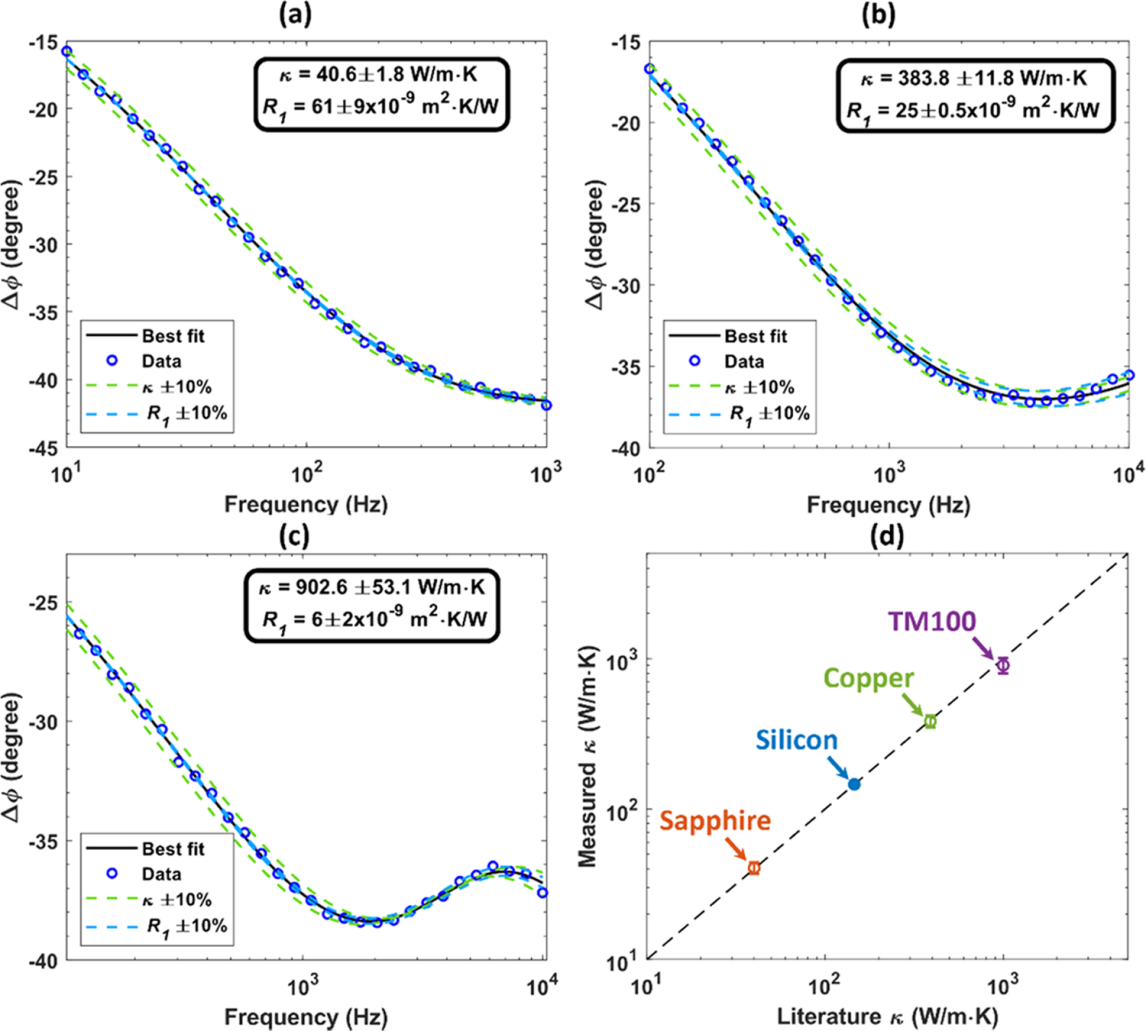
Measured phase data and best-fit curves for (a) sapphire, (b) copper, and (c) TM100 samples along with model curves when the fitted thermal conductivity κ and thermal boundary resistance *R*_1_ are varied by ±10%. (d) Extracted thermal conductivities for copper, sapphire, and TM100 samples (*y*-axis) versus their corresponding literature values (*x*-axis).

A range of bulk materials relevant to electronic devices and packaging were measured, spanning a ∼30× range of thermal conductivities, to verify the instrument’s accuracy: sapphire, silicon, copper, and CVD diamond (TM100). Figure 4 shows the best-fit thermal conductivities for each sample, using the fixed, known densities and specific heat capacities given in Table 1; *R*_1_ is also fitted, even though its sensitivity is relatively low, as shown in Figure 2. The correlation between measured and known thermal conductivity values is plotted in Figure 4d, showing a deviation of only 1.6, 1.4, and 9.7% for the sapphire, copper, and TM100 samples, respectively, confirming the measurement accuracy over a wide range of thermal conductivities. The deviation between measured and datasheet thermal conductivity of the diamond sample is comparable to the measurement error bar but higher than that of the other materials due to the high thermal conductivity of diamond, which reduces the relative signal amplitude. Nevertheless, it would be impossible to measure diamonds of this thickness using standard laser flash, which emphasizes the versatility of our method. The measurement accuracy of individual layers gives confidence in the accuracy of multilayer structure measurements, where the thermal properties of some or all of the layers are unknown.

### Multilayer Structure Measurements

The previous section focused on FDTR measurement of bulk materials to verify the accuracy of the instrument and the data analysis method. In this section, we demonstrate the suitability of low-frequency FDTR for measuring the thermal properties of individual layers of a multilayer structure in situ. The suitability of this technique is demonstrated here, for example, on a GaN transistor die mounted on a copper flange using a sintered silver as a die attach, as illustrated in Figure 1a. The silver-sintered die attach is the layer of interest here since the properties of the other layers are known. The silver-sintered die attach is from a commercial supplier with a datasheet thermal conductivity of ∼200 W/m·K. This is a bulk material thermal conductivity measured by LFA. Bulk thermal conductivity values, such as measured by laser flash, ignore potential thermal resistances at the SiC/die attach and die attach/copper interfaces, including contributions from the substrate metallization layers and the intrinsic thermal boundary resistance between dissimilar materials. Void formation close to interfaces is also possible, which increases the thermal resistance of the die attach layer.^19^

**Figure 5:**
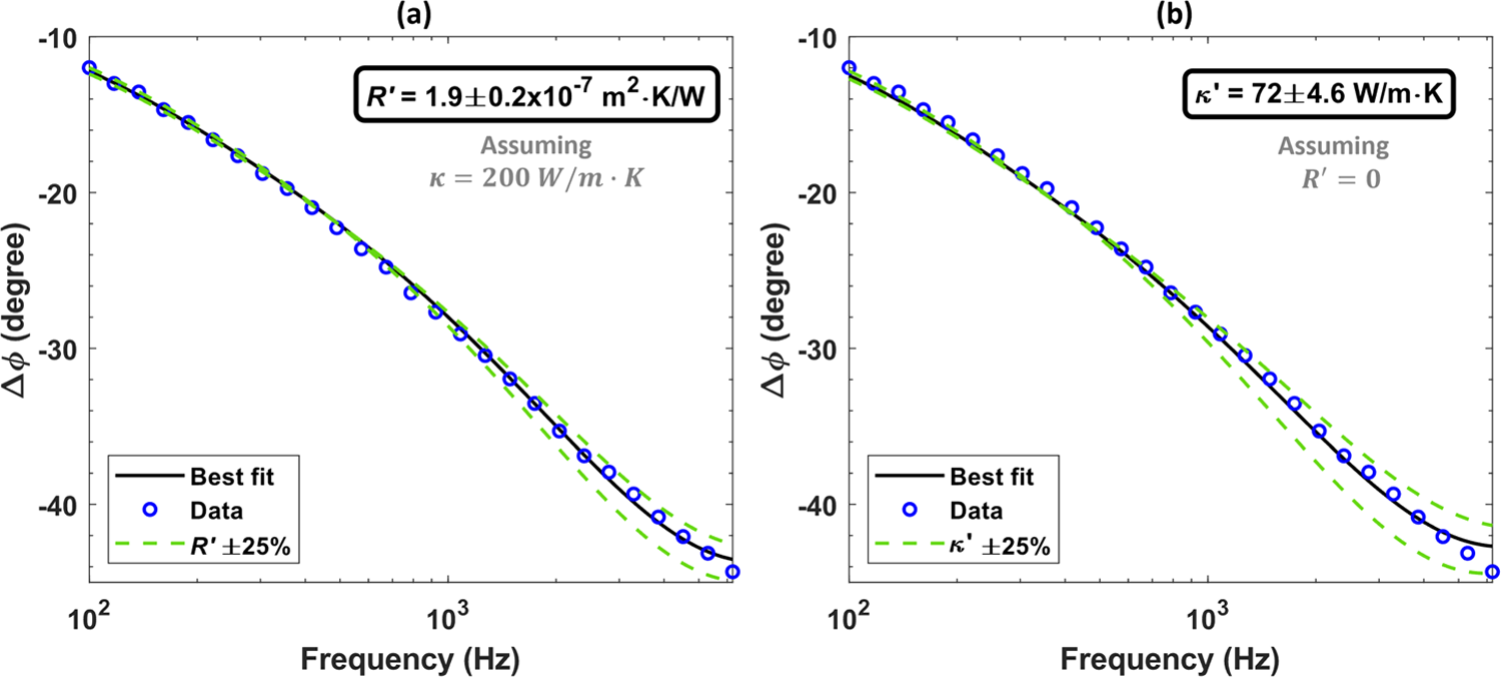
Measured phase data and best-fit curves for the die attach sample under study. Two different fitting parameters have been considered. (a) Thermal boundary resistance *R*′ = *R*_3_ + *R*_4_. (b) Effective thermal conductivity of the die attach (κ′).

Figure 5 shows the results fitted for two scenarios: (a) assuming the manufacturer’s bulk thermal conductivity and fitting the interface thermal resistance *R*′ = *R*_3_ + *R*_4_; (b) assuming that there is no interface thermal resistance and fitting the effective thermal conductivity of the die attach layer. Both scenarios produce an equally good fit, indicating that the contribution of silver-sintered layer thermal conductivity and interface thermal resistances are indistinguishable, but the die attach bulk and effective (in situ) thermal conductivity differ by 2.9×. Fixed thermal boundary resistances at the gold transducer/GaN interface (*R*_1_) and at the GaN/SiC interface (*R*_2_) values of 1 × 10^–9^ m^2^·K/W and 2 × 10^–8^ m^2^·K/W were used based on previous measurements,^38^ respectively.

**Figure 6:**
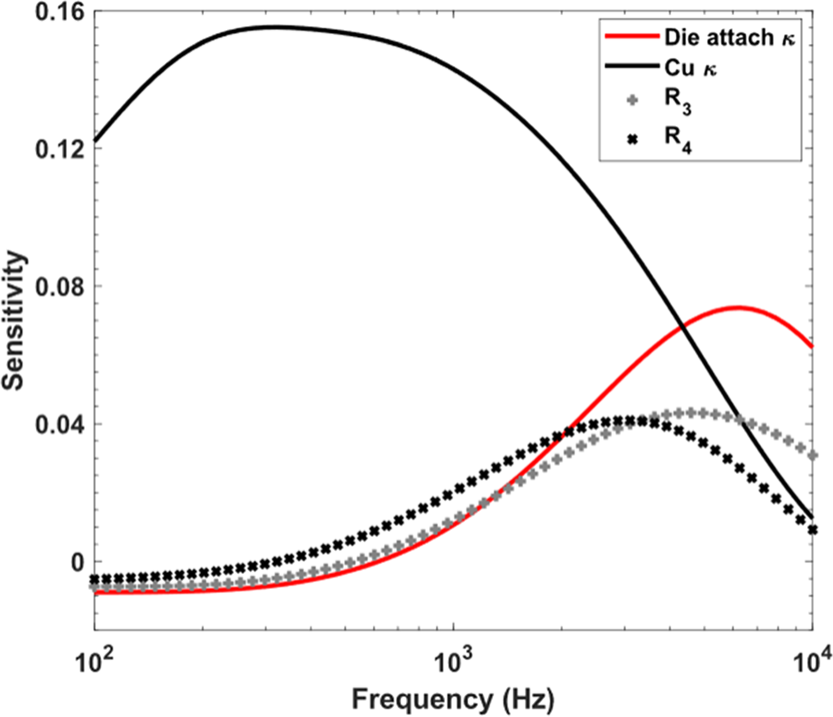
Die attach and copper thermal conductivity (κ) phase sensitivities, also showing the thermal boundary resistance of the SiC/die attach (*R*_3_), and die attach/copper (*R*_4_) interfaces. The parameters used are 200 W/m·K for die attach thermal conductivity and *R*_3_ = *R*_4_ = 1 × 10^–7^ m^2^·K/W.

Figure 6 represents the phase sensitivity to the die attach and copper heat sink thermal conductivity, the thermal boundary resistances at the SiC/die attach interface (*R*_3_) and at the die attach/copper interface (*R*_4_). The properties used in the sensitivity study are listed in Table 2, and the initial thermal conductivity for the silver-sintered die attach is 200 W/m·K, and the initial value for *R*_3_ and *R*_4_ is 1 × 10^–7^ m^2^·K/W (considering a typical value based on previous measurements). The sensitivity plots confirm that the frequency response of the die attach thermal interface resistances, upper and lower, and the thermal conductivity of the 20 μm-thick sintered silver layer (peak sensitivity ∼6kHz) are similar. The copper heat sink has a much lower frequency peak sensitivity at 300Hz and can be clearly distinguished independently in the measurement.

**Table 2:** Known Properties of Die Attach Sample^a^

layer	*d* (μm)	κ (W/m·K)	ρ (g/cm^3^)	*c*_p_ (J/g·K)
GaN	2	156	6.15	0.490
SiC	75	420	3.21	0.648^39^
die attach	20	72–200^b^	8.58	0.233^40^
Cu	1000	390	8.94	0.386

a . The properties include thickness (*d*), thermal conductivity (κ), density (ρ), and specific heat capacity (*c*_p_)

b Fitted versus manufacturer’s values.

We therefore use a similar approach to ref (19), and the total thermal resistance of the die attach layer can be expressed as
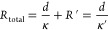
9where *R*_total_ is the total thermal resistance of the die attach in m^2^·K/W, *d* is the thickness of the die attach layer, *R*′ = *R*_3_ + *R*_4_ is the total interface thermal resistance, and κ′ is a lumped term representing the effective die attach thermal conductivity. From eq 9, *R*_total_ = 2.78 × 10^–7^ m^2^·K/W and *R*_total_ = 2.8 × 10^–7^ m^2^·K/W are obtained from the fits shown in Figure 5a,b, respectively, which are identical, considering the measurement uncertainty. This shows that fitting a lumped thermal resistance or fitting each contribution independently produces the same total thermal resistance for the die attach layer. However, using the provided bulk thermal conductivity of 200 W/m·K and ignoring the interface thermal resistance is equivalent to a die attach thermal resistance of 1 × 10^–7^ m^2^·K/W. Underestimating the actual measured total thermal resistance of the die attach layer by ∼3× highlights the importance of in situ measurement.

**Figure 7:**
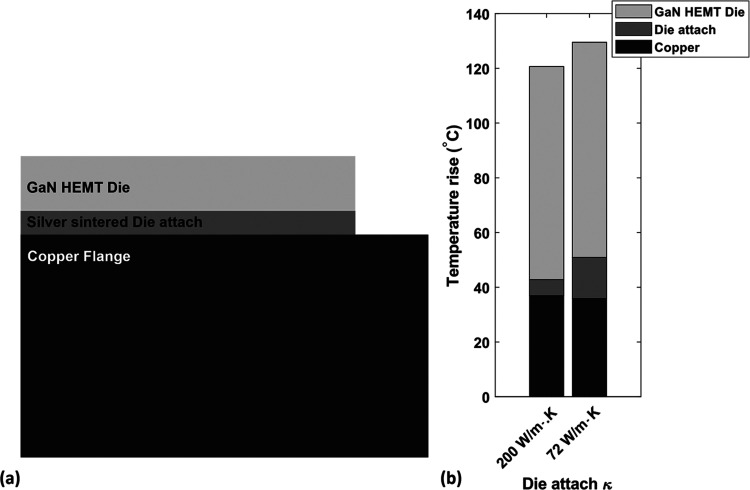
(a) Two-dimensional (2D) schematic representation of the 3D FE thermal model of a Wolfspeed CGH40010 packaged GaN HEMT. The die attach layer is 20 μm thick, and the flange is pure copper. (b) Simulated temperature rise across the die, die attach, and the flange using the bulk die attach value of 200 W/m·K and the measured effective fitted conductivity of 72 W/m·K. The power dissipation was fixed at 5 W/mm and at a baseplate temperature of 80 °C.

Practically, the total thermal resistance associated with the die attach is needed to estimate the channel temperature of an electronic device. Figure 7a illustrates a three-dimensional (3D) finite element (FE) thermal model of a commercial packaged GaN-on-SiC transistor (Wolfspeed CGH40010). The GaN HEMT die material parameters and model accuracy have been checked using Raman thermography measurements, described in detail in ref (41). In the thermal model, the die is mounted on a pure copper flange using a 20 μm silver-sintered die attach. Figure 7b represents the simulated temperature rise across the die, die attach, and the flange for two different die attach thermal conductivity values: bulk vendor’s value of 200 W/m·K (excluding interface thermal resistance) and the measured effective thermal conductivity value of 72 W/m·K. By assuming the die attach vendor’s thermal conductivity, the peak channel temperature rise is underestimated by ∼7% with respect to the correct value based on the actual effective die attach thermal conductivity, which would severely affect reliability.^42^

### Sensitivity Study for Different Multilayer Structures

**Figure 8:**
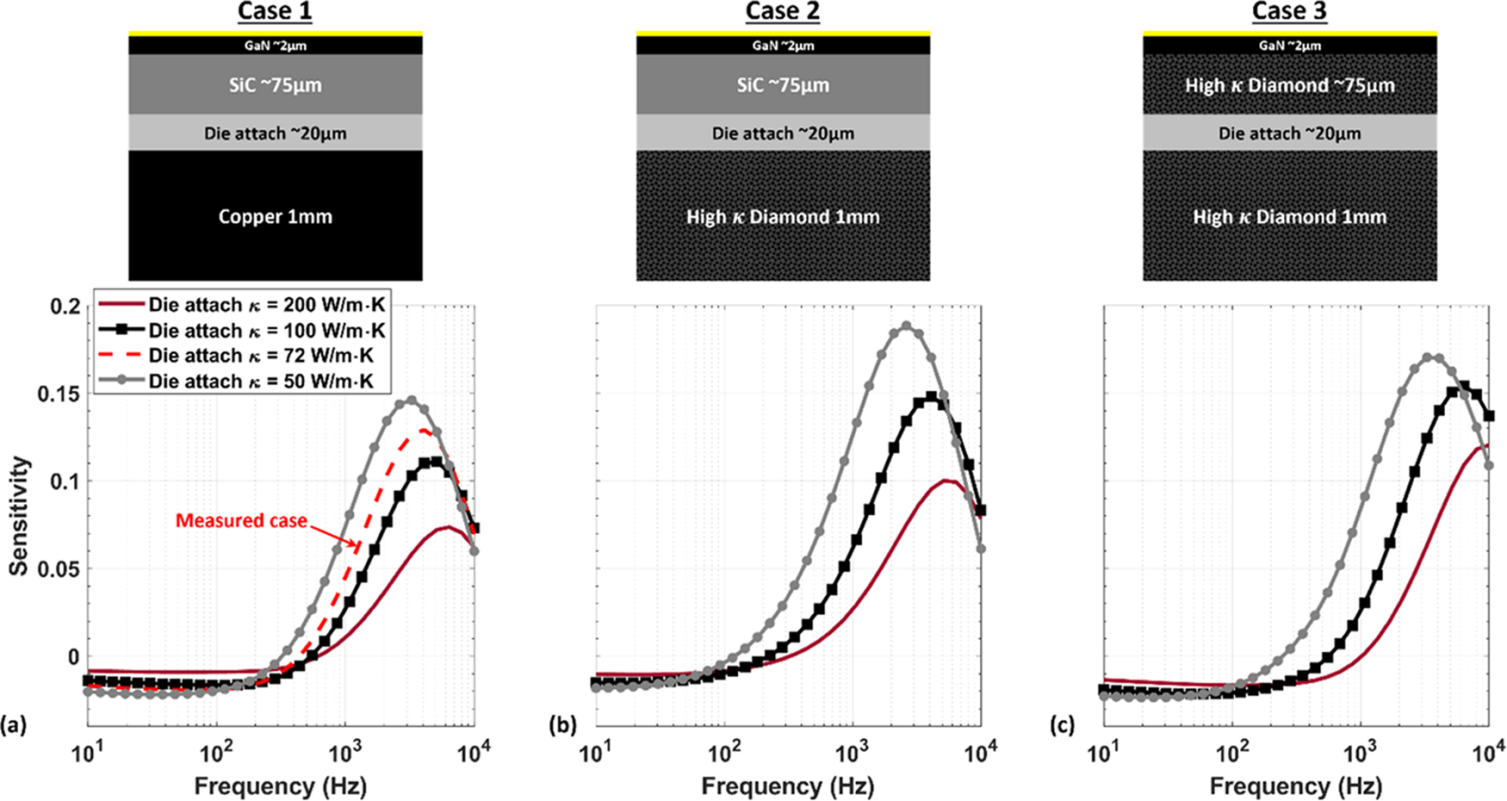
Case 1: (a) Reflected phase sensitivities to different die attach thermal conductivity; (a) Case 1, (b) Case 2, and (c) Case 3. The structures are presented above the sensitivity study.

Measurement of a packaged GaN-on-SiC transistor was demonstrated in the previous section, which corresponds to Case 1 in Figure 8a. In this section, the sensitivity of the reflected phase to the die attach thermal conductivity (κ) is studied analytically for two additional multilayer structures (Figure 8a–c). The first observation from this sensitivity study is that by lowering the die attach κ, the peak sensitivity shifts to lower frequencies, along with an increase in the sensitivity amplitude. Second, by replacing the copper heat sink in Case 1 with a 1000 W/m·K diamond heat sink (Case 2), the phase sensitivity amplitude increases by a factor of ∼1.3, and the peak sensitivity shifts slightly to lower frequencies. Finally, by replacing the SiC substrate in Case 2 with higher κ material substrate (e.g., diamond), the peak sensitivity of the die attach (Figure 8c), in case of a 200 W/m·K, is at the upper limit of the measurement frequency range (10 kHz). The high sensitivity to the die attach layer for a range of the substrate, die attach, and heat sink thermal conductivities demonstrates the versatility of the low-frequency FDTR technique to measure different material combinations.

## Conclusions

A versatile low-frequency FDTR measurement technique tailored to measure the thermal properties of multilayer or bulk samples, with thickness ranging from tens of micrometers to millimeters is demonstrated. This is essential for optimizing the next generation of low thermal resistance electronics packaging and heat spreaders, which are needed for high power density devices. The accuracy of the technique was demonstrated on a range of reference materials, including through a 0.25 mm-thick CVD diamond, which has previously been difficult to measure with high accuracy using existing standard techniques. The capability to measure through multilayer samples with depth resolution was demonstrated on the example of a GaN-on-SiC chip mounted on a copper flange using silver-sintered die attach. The measured effective thermal resistance of the die attach results in a 7% higher predicted transistor temperature compared to using the bulk thermal conductivity value, which highlights the importance of accurate in situ thermal conductivity analysis.
